# Diversity of gut microflora is required for the generation of B cell with regulatory properties in a skin graft model

**DOI:** 10.1038/srep11554

**Published:** 2015-06-25

**Authors:** R. Alhabbab, P. Blair, R. Elgueta, E. Stolarczyk, E. Marks, P. D. Becker, K. Ratnasothy, L. Smyth, N. Safinia, E. Sharif-Paghaleh, S. O’Connell, R. J. Noelle, G. M. Lord, J. K. Howard, J. Spencer, R. I. Lechler, G. Lombardi

**Affiliations:** 1 Medical Research Council Centre for Transplantation, King’s College London, King’s Health Partners, Guy’s Hospital, London SE1 9RT, UK; 2 Division of Diabetes and Nutritional Sciences, King’s College London, Guy’s Hospital, London SE1 9RT, UK; 3 Peter Gorer Department of Immunobiology, King’s College London, Guy’s Hospital, London SE1 9RT, UK

## Abstract

B cells have been reported to promote graft rejection through alloantibody production. However, there is growing evidence that B cells can contribute to the maintenance of tolerance. Here, we used a mouse model of MHC-class I mismatched skin transplantation to investigate the contribution of B cells to graft survival. We demonstrate that adoptive transfer of B cells prolongs skin graft survival but only when the B cells were isolated from mice housed in low sterility “conventional” (CV) facilities and not from mice housed in pathogen free facilities (SPF). However, prolongation of skin graft survival was lost when B cells were isolated from IL-10 deficient mice housed in CV facilities. The suppressive function of B cells isolated from mice housed in CV facilities correlated with an anti-inflammatory environment and with the presence of a different gut microflora compared to mice maintained in SPF facilities. Treatment of mice in the CV facility with antibiotics abrogated the regulatory capacity of B cells. Finally, we identified transitional B cells isolated from CV facilities as possessing the regulatory function. These findings demonstrate that B cells, and in particular transitional B cells, can promote prolongation of graft survival, a function dependent on licensing by gut microflora.

There is a body of evidence that B cells can contribute to allograft rejection^1^,^2^,^3^,^4^,^5^. In mice, depletion of B cells has been shown to delay renal allograft rejection, and in humans the involvement of B cells in promoting graft rejection has been suggested by the beneficial effects of B cell depletion therapy (Rituximab) for kidney transplant recipients^3^,^6^,^7^. However, there is now also evidence to suggest that B cells may have a role in promoting tolerance to allografts. One study using Rituximab as induction therapy for kidney transplants found that the depletion of B cells led to acute cellular rejection in some patients, suggesting that B cells may contribute to allograft survival by restraining allo-immune responses^8^. We have recently reported that immunosuppressive drug free transplant patients who had become spontaneously tolerant to their HLA mismatched kidney transplants had elevated numbers of peripheral blood B cells and upregulated expression of several genes associated with B cell function^9^. Similarly, Newell *et al.* have shown that drug free tolerant patients had a higher proportion of transitional B cells in their peripheral blood compared to non-tolerant patients and similar levels to healthy controls, results that were confirmed by Silva *et al.*^10^,^11^. Further, patients with chronic graft versus host disease following allogeneic stem cell transplantation have reduced regulatory B cell numbers. However mesenchymal stem cell treatment restores Breg function, a feature which correlates with an improved clinical outcome^12^,^13^. In mice, there are now multiple reports that implicate a regulatory role for B cells in graft survival^14^,^15^,^16^,^17^,^18^,^19^,^20^.

In autoimmune models, two major phenotypes of regulatory B cells have been described, CD5^+^CD1d^hi^ B10 B cells and CD19^+^CD21^hi^CD23^hi^CD24^hi^ transitional-2 (T2) regulatory B cells^21^. Both have been shown to contribute to the suppression of inflammation in a number of mouse autoimmune models. For example, adoptive transfer of T2 B cells can inhibit collagen induced arthritis (CIA) and lupus^21^. In all these studies IL-10 was demonstrated to be the major mediator of their function^22^,^23^. Interestingly, Shimumura *et al.* reported that the degree of sterility in which mice are housed, could alter the function of regulatory B cells. B cells could regulate chronic colitis only when the mice were housed under non-hygienic conditions^24^. More recently Rosser *et al.* demonstrated that regulatory B cells had reduced ability to prevent experimental arthritis when isolated from mice under sterile specific pathogen free (SPF) compared to regulatory B cells isolated from mice in less sterile conventional (CV) housing. Ablation of gut microflora with antibiotics treatment further reduced regulatory B cell ability to inhibit arthritis development^25^.

Here, we use a mouse model of MHC-class I mismatched skin transplantation to investigate whether sterility of housing influences B cell ability to prolong skin graft survival. Adoptive transfer of B cells isolated from naïve SPF mice did not prolong skin transplant survival and their lack of regulatory function was confirmed *in vitro*. However, when B cells were isolated from naïve mice that had been kept under less hygienic conventional (CV) conditions, adoptive transfer of B cells prolonged skin graft survival and suppressed TNF-α production by T cells *in vitro*. In contrast, transfer of B cells from IL-10 deficient mice housed in CV facilities did not prolong skin graft survival. Also, the regulatory function of B cells was abolished by treatment of the mice with antibiotics. Finally, we identified that within total B cells the transitional B cell subtypes were the subpopulations carrying the regulatory function. These results demonstrate that B cells, and in particular transitional B cells, acquire a regulatory role when the mice are kept under non-hygenic condition and this lead to an increase in skin graft survival.

## Results

### B cells isolated from mice kept in low sterility conventional facilities prolong MHC-class I mismatched skin graft survival

**Figure 1:**
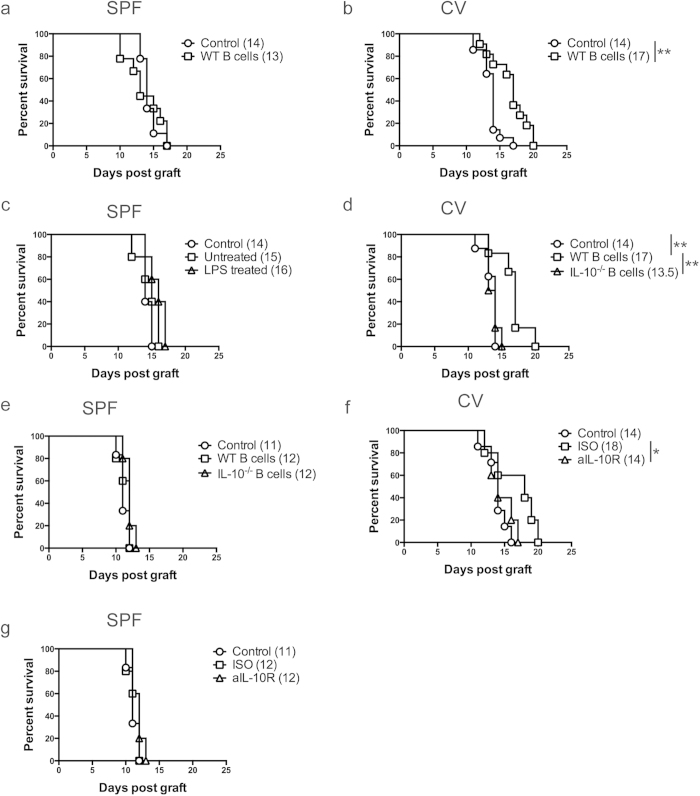
Adoptive transfer of B cells isolated from mice kept in CV facilities can prolong MHCI mismatched skin graft survival. CD8 depleted B6 (H2-K^b^) mice received dorsal skin grafts from B6-K^d^ (H2-K^d^) mice. One day prior to skin grafts mice received i.v. injections of 1 × 10^7^ B cells that were FACS purified from naïve B6 mice. Controls received PBS. Plots show skin graft survival when graft and cell transfer are performed on mice kept in (**a**) SPF facilities (n ≥ 9/group) and (**b**) CV facilities. (n ≥ 11/group). (**c**) B cells from naïve B6 mice maintained in SPF facilities were treated (LPS treated) or not (Untreated) with 2 μg/mL LPS for 16 hrs. Then, 1 × 10^7^ B cells were adoptively transfer one day prior to skin grafts, controls received PBS. The mice were kept in SPF facilities (n ≥ 5/group). (**d**,**e**) Injections of 1 × 10^7^ B cells that were FACS purified from IL-10^−/−^ or WT naïve B6 mice kept in CV (**d**) or SPF (**e**) facilities. Controls received PBS. The adoptive transfer was made one day prior to skin grafts. Plots show skin graft survival when graft and cell transfer are performed on mice kept in CV (**d**) or SPF (**e**) facilities (n ≥ 6/group for each group). (**f**,**g**) Plots show skin graft survival when cell transfer of 10^7^ B cells from mice treated with anti-IL10R (aIL-10R) antibody or isotype control (ISO) is performed on mice kept in CV (**f**) or SPF (**g**) facilities (n ≥ 6/group for each group). Mean survival in days is denoted between parentheses. Statistics were calculated by log-rank (Mantel-Cox) test + Bonferonni correction for multiple comparisons. ^*^p < 0.05, ^**^p < 0.01.

It has previously been reported that the adoptive transfer of B cells suppresses the development of autoimmunity in several murine models^22^,^23^,^26^ and that B cells may have a regulatory function in inducing graft survival^14^,^15^,^16^,^17^,^18^,^19^,^20^. In this study, the capacity of B cells to prolong skin transplant survival was investigated. B cells (1 × 10^7^) enriched by negative selection (>95% purity) from the spleens of naïve C57BL/6 (B6) mice housed in high sterility specific pathogen free (SPF) facilities were adoptively transferred intravenously (i.v.) to naïve B6 mice. One day following adoptive transfer B6 mice (H-2^b^) received dorsal skin transplants from MHC I mismatched transgenic B6 mice that express MHC-I H-2K^d^ (B6-K^d^). CD8^+^ T cells were depleted by intraperitoneal (i.p.) injection of anti-CD8 antibody on day −1, 0 (day of skin graft), +1 and every 7 days following skin transplant, to eliminate the contribution of CD8^+^ T cells with direct allospecificity to graft rejection, as previously published^27^. The results in Fig. 1a demonstrate that skin graft survival was not prolonged by the adoptive transfer of B cells in SPF facilities.

In a previous publication it was shown that the ability of B cells to inhibit chronic colitis, or experimental arthritis, depended on the sterility of the facilities within which the animals were housed^25^,^28^. We thus tested whether B cells isolated from mice housed in less sterile conventional (CV) facilities were able to prolong graft survival. Figure 1b demonstrates that adoptive transfer of 10^7^ B cells isolated from mice housed in CV facilities significantly prolonged the survival of B6-K^d^ skin grafts compared to control mice. The prolongation of skin graft survival observed when B cells were transferred was of similar extent to the transfer of 5 × 10^6^ graft-specific regulatory T cells (Tregs) in the same strain combination^27^.

Given that the bacterial component lipopolysaccharide (LPS) has been suggested to be important for the generation of regulatory B cells^29^,^30^, and that altering the sterility of housing may affect the amount of LPS to which B cells are exposed *in vivo*, we investigated whether incubation of SPF B cells with LPS could restore their regulatory function. B cells from mice housed in the SPF facility were pre-incubated *in vitro* with LPS for 16 hours before adoptive transfer. Figure 1c shows that adoptive transfer of 10^7^ LPS treated SPF isolated B cells to B6 mice kept in SPF facilities was able marginally to delay graft rejection of B6-K^d^ skin grafts compared to control mice, however the difference did not reach statistical significance. This result suggests that increased exposure to LPS stimulation alone does not explain the enhanced regulatory function displayed by B cells isolated from CV facilities and that other factors are involved.

IL-10 has been shown to be the key cytokine produced by regulatory B cells in autoimmune models^21^,^22^. However, in animal models of graft rejections the role of IL-10 produced by B cells in prolonging graft survivals has been more controversial^16^,^18^,^19^,^20^,^31^. To test directly whether IL-10 plays any role in the regulatory function of B cells, B cells were isolated from IL-10 deficient mice housed in either CV facilities (Fig. 1d) or in SPF facilities (Fig. 1e) and their ability to prolong graft survival in either facility was compared to B cells from WT mice. Prolongation of skin graft survival was not observed following transfer of IL-10^−/−^ B cells (Fig. 1d,e) isolated from mice kept in either facility.

These results in Fig. 1d,e suggest that IL-10 production by B cells is important for the B cell mediated prolongation of skin graft survival observed in CV facilities. However the complete lack of IL-10 in IL-10-deficient B cell donor mice might in fact be inhibiting the development of regulatory B cells. To investigate this possibility, IL-10 competent B6 mice housed in CV (Fig. 1f) or SPF (Fig. 1g) facilities were injected with a neutralizing antibody specific for the IL-10 receptor (aIL-10R) or with the isotype antibody (ISO) for two weeks. B cells were then purified from these animals and transferred to mice the day before receiving skin grafts. We observed that the treatment of mice with aIL-10R antibody abolished the prolongation of skin transplant survival obtained with B cells derived from the ISO group in CV facilities (Fig. 1f). Blockade of IL-10R did not alter the lack of regulatory function observed in B cells isolated from mice kept in SPF facilities (Fig. 1g). These results together suggest that IL-10 appears to be necessary for the development of regulatory B cells *in vivo*, however it does not excluded the possibility that IL-10 is also necessary for B cell regulatory function.

### Mice housed under non-hygienic conditions have an activated adaptive immune system

**Figure 2:**
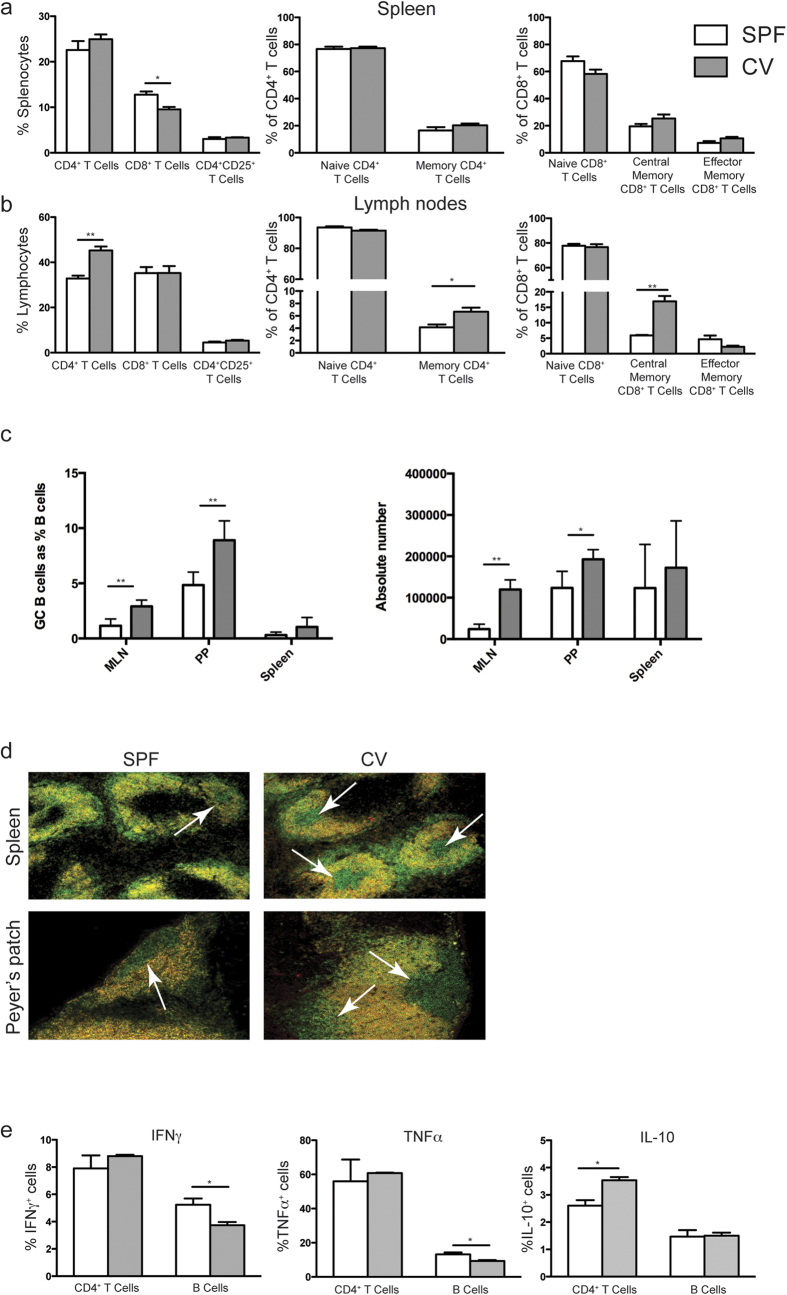
Immunological differences between mice kept in SPF & CV facilities. Spleens, lymph nodes & PPs were collected from B6 mice (6 weeks old) maintained in SPF and CV facilities, and phenotyped for B cell and T cell populations by using antibodies against CD4, CD8, CD25, CD19, GL7, CD38, CD95, CD44, & CD62L. (**a**) and (**b**) Histograms show mean percentage ± SEM CD4^+^, CD8^+^ and CD4^+^CD25^+^ T cells in total splenocytes or total lymph node cells, naïve (CD64L^hi^CD44^-^) and memory (CD62L^hi^CD44^+^) CD4^+^ T cells as percentages of total CD4^+^ T cells, and naïve and memory CD8^+^ T cells as percentages of total CD8^+^ T cells in (**a**) Spleens and (**b**) Lymph nodes. (**c**) Histograms show the mean ± SEM of the percentages of GC B cells (GL7^high^ and CD95^+^, left graph) and absolute number (right graph) in the spleens, MLN and PPs of SPF and CV mice. (**d**) Immunofluorescence microscopy pictures of B220 and IgD stained frozen sections from spleens (top row) and PPs (bottom row) of mice kept in SPF and CV facilities. Pictures show B220 (green) and IgD (Red). Germinal centres are marked by arrows. (**e**) Histograms show mean ± SEM of IFNγ, TNFα, and IL-10 expression by splenic CD4^+^ T cells and B cells following 5 hrs *in vitro* stimulation with PMA & ionomycin. n = 3. Statistics were calculated by t test, ^*^P < 0.05 & ^**^P < 0.005.

Next, we investigated whether there were any immunological differences between mice kept in CV and SPF facilities that might explain the difference in the capacity of B cells to prolong skin graft survival. Spleens and lymph nodes (LN) were collected from mice maintained in both facilities, and the proportions of B and T cell subsets were analysed *ex vivo*. No differences were found in total splenic B cells, however, there was a lower percentage of total B cells in the LN of mice maintained in CV facility compared to mice maintained in the SPF facility (Fig. S1a and S1b). Additionally, CV mice displayed significantly higher percentages of total CD4^+^, memory (CD62L^hi^CD44^+^) CD4^+^ and central memory CD8^+^ T cells in peripheral LN compared to SPF mice, while lower percentages of CD8^+^ T cells were detected in the spleens of B6 mice maintained in SPF facility compared to mice from the CV facility were observed (Fig. 2a,b). These results suggest an alteration in the B cell:T cell ratio between the LN of animals housed in CV and SPF facilities.

Splenocytes, LN, mesenteric LN (MLN) and Peyer’s patch (PP) cells were stained for the expression of CD19, GL-7 and CD95 and germinal centre (GC) B cells were identified by flow cytometry. As shown in Fig. 2c mice housed in CV facilities had significantly higher frequencies and absolute numbers of GC B cells in the MLNs and PPs compared to mice kept in SPF facilities. Moreover, there were higher frequencies and absolute numbers of GC B cells in the spleens of mice housed in CV facilities, however the differences were not statically significant (Fig. 2c). The presence of higher numbers of GCs in the spleens of CV mice compared to SPF mice was confirmed by confocal microscopy as Fig. 2d shown.

Having demonstrated differences in the percentages of memory T cells and in the levels of GC B cells between mice kept in the CV and SPF, cytokine expression by B and T cells was investigated. IFN-γ, IL-10 and TNF-α expression by splenic B and T cells isolated from CV and SPF mice was assessed by intracellular staining following 6 hrs stimulation with PMA and ionomycin. The results in Fig. 2e show that B cells isolated from mice maintained in CV facilities expressed significantly lower levels of IFN-γ and TNF-α compared to those isolated from SPF facilities, with no differences in the production of IL-10. T cells obtained from mice kept in CV facilities expressed significantly higher levels of IL-10 compared to those isolated from mice maintained in SPF facilities with no major differences in TNF-α and IFN-γ (Fig. 2e). These results suggest that mice housed in less hygienic conditions have a bias towards a more anti-inflammatory microenvironment in the spleen and that both B cells and T cells from mice maintained in the CV facilities have a trend to an higher IL-10/TNF-α ratio compared to the same cell types from mice housed in the SPF facilities.

### Mice kept in CV facilities have an altered composition of gut microbiota compared mice housed in the SPF facilities

**Figure 3:**
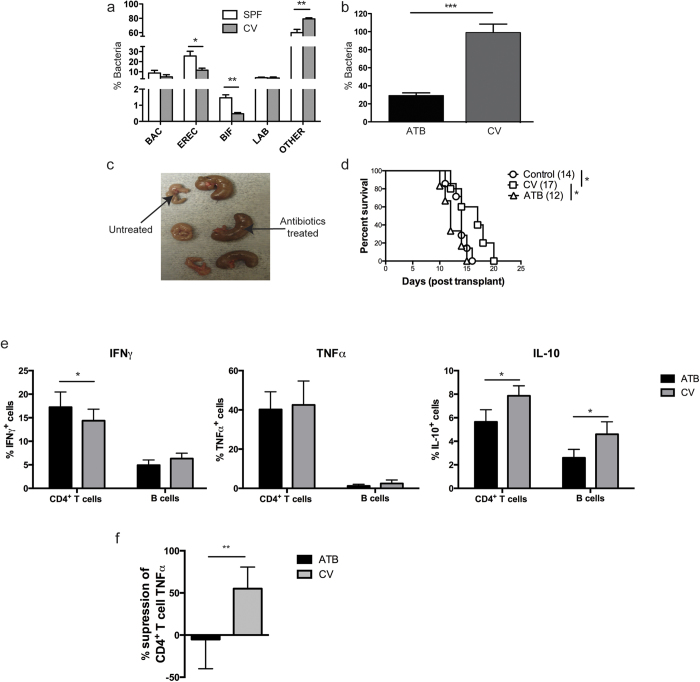
Mice kept in CV facility have different composition of microbiota than mice housed in SPF facility. Faecal samples were collected from B6 mice kept in SPF & CV facilities, or kept in CV facility and treated with antibiotics for 2 weeks. Different bacterial groups (Bac = *Bacteroides* and *Prevotella*, Erec = *Lachnospiraceae*, Bif = *Bifidobacterium*, Lab = *Lactobacillus* & *Streptococcus*) were identified by FISH-Flow. Histograms show mean ± SEM for (**a**) proportions of the dominant fecal microbiota in B6 mice maintained in SPF and CV facilities, and (**b**) ratio of total microbiota in antibiotic treated (ATB) and non-treated (CV) B6 mice maintained in CV facilities (the mean total bacteria in untreated mice is defined as 100%). (**c**) Pictures of caeca recovered from antibiotics treated and non-treated B6 mice maintained in CV facilities. (**d**) CD8 depleted B6 (H2-K^b^) mice received dorsal skin grafts from B6-K^d^ (H2-K^d^) mice. One day prior to skin grafts mice received i.v. Injections of 1 × 10^7^ B cells that were FACS purified from antibiotics (ATB) or control (CV) naïve B6 mice kept in CV facilities. Plots show skin graft survival when graft and cell transfer are performed on mice kept in SPF facilities. n ≥ 6/group for each group. Mean survival in days is denoted between parentheses. (**e**) Histograms show mean ± SEM of IFNγ, TNFα, and IL-10 expression by splenic CD4^+^ T cells and B cells from mice treated with antibiotics (ATB) or non-treated (CV) naïve B6 mice kept in CV facilities, 2 independent experiments with 10 mice per group. (**f**) B cells were isolated from spleens of B6 mice treated with antibiotics or not and maintained in CV facilities. B cells were co-cultured with CD4^+^ T cells and irradiated allo-DCs (25 CD4 T cells:25 B cells:1 allo-DC) for 48 hrs. Summary data showing percentage of TNFα supression by B cell from spleens of B6 mice antibiotics treated or non-treated and housed in CV facilities. Histograms display mean ± SEM, 2 independent experiments with 10 mice per group. Statistics were calculated by log-rank (Mantel-Cox) test + Bonferonni correction for multiple comparisons and t test, ^*^P < 0.05, ^**^P < 0.005 & ^***^P < 0.001.

Previous reports have shown that the presence of commensal microbiota affects the development of immune responses and can protect humans from atopic diseases^32^,^33^. In addition, gut microbiota have been shown to influence B cell secretion of immunoglobulin, and support the development of splenic regulatory B cells, suggesting that the presence of bacteria in the gut has a measurable impact on B cell function^25^,^34^. Having identified differences in the general activation status of the B and T cells of the immune system between mice housed in the SPF and CV facilities, and in the regulatory potential of B cells from CV and SPF mice, we analysed faecal samples to determine whether these differences were associated with differences in their gut microbiota. The data shown in Fig. 3a demonstrates that the composition of the gut microbiota was significantly different between mice housed in CV and SPF facilities. Mice maintained in CV facilities displayed a reduction in the proportions of *Lachnospiraceae* (Erec 482^+^) and *Bifidobacterium* (Bif 164^+^) groups compared to mice maintained in SPF facilities. To investigate the role of bacteria in the induction of regulatory B cell function, mice housed in CV facilities were treated for 2 weeks with broad-spectrum antibiotics. The efficacy of the antibiotic treatment was corroborated by a reduction of faecal microflora content by 70% in the colonic content of antibiotic treated compared to non-treated mice (Fig. 3b). It has been previously reported that caecal size increases in response to augmented hydrated dietary fibre components following a reduction in the bacterial load^35^. Here, mice treated with antibiotics displayed a marked increase in caecum size confirming the efficacy of the treatment (Fig. 3c). We thus tested whether B cell isolated from antibiotic treated mice housed in the CV facilities were able to prolong graft survival. Figure 3d shows that, unlike adoptive transfer of control B cells from non-antibiotic treated mice, adoptive transfer of 10^7^ B cells isolated from antibiotic treated mice housed in CV facilities was unable to prolong the survival of B6-K^d^ skin grafts. All these results together suggest that the intestinal microbiota is essential to maintain the capacity of B cells to delay skin graft rejection.

We also evaluated whether the antibiotic treatment had an effect on the cytokine production of B and T cells. IFN-γ, IL-10 and TNF-α expression by splenic B and T cells isolated from CV housed mice treated with antibiotics, or untreated controls, was assessed by intracellular staining following 4 hrs stimulation with PMA and ionomycin. The results in Fig. 3e show that T cells isolated from mice treated with antibiotics expressed significantly higher levels of IFN-γ compared to those isolated from control mice. Both, B and T cells obtained from mice treated with antibiotics expressed significantly lower levels of IL-10 compared to those isolated from control mice (Fig. 3e). To determine whether the differences in cytokine production between B cells from antibiotics treated and untreated mice reflected a difference in regulatory capacity, B cell subsets were tested *in vitro* for the ability to suppress allo-specific T cell activation. B cells have been shown previously in autoimmune models to suppress cytokine production by CD4^+^ T cells *in vitro*, in particular the expression of TNF-α^23^. B cell were purified from the spleens of CV facility housed B6 mice that had either been treated with antibiotics or from untreated controls, and co-cultured with CD4^+^ T cells from spleen of SPF facility housed B6 mice, that had been stimulated by irradiated H2-K^d^ expressing allogeneic dendritic cells (allo-DCs) for 48 hours. Our results show that following stimulation of CD4^+^ T cells with allo-DC, B cells from the spleens of B6 mice treated with antibiotics lost the ability to suppress TNF-α expression by CD4^+^ T cell (Fig. 3f). Therefore, we suggest that microbiota is involved directly in empowering B cells to suppress T cell responses.

### Transitional B cells isolated from mice kept in low sterility conventional facilities prolong MHC-class I mismatched skin graft survival and suppress allospecific TNF-α expression by CD4^+^ T cell *in vitro*

**Figure 4:**
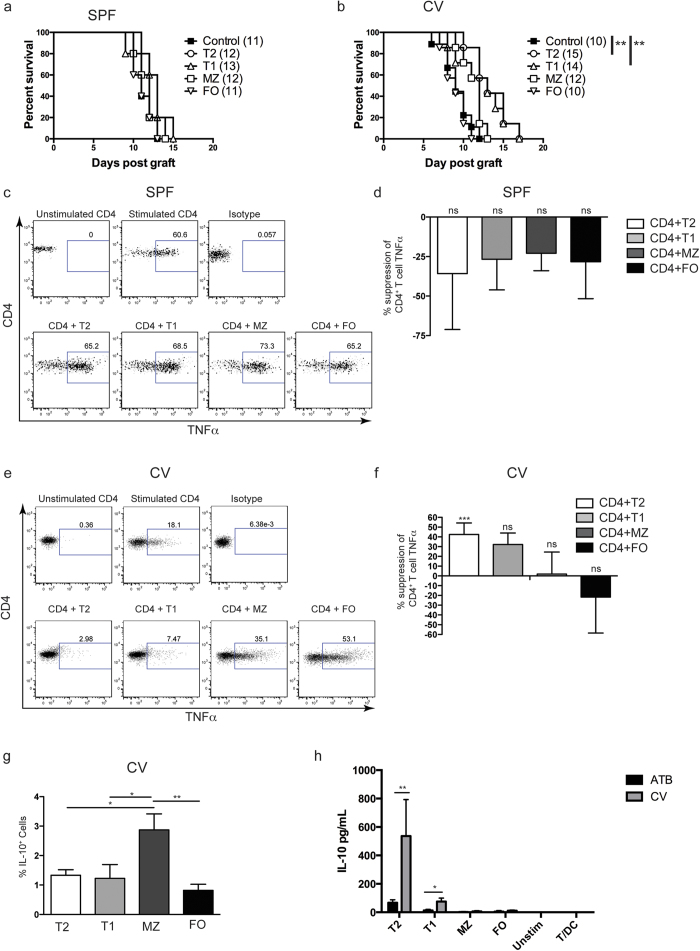
Adoptive transfer of T2 B cells isolated from mice kept in CV facilities can prolong MHC I mismatched skin graft survival. CD8 depleted B6 (H2-K^b^) mice received dorsal skin grafts from B6-K^d^ (H2-K^d^) mice. One day prior to skin grafts mice received i.v. Injections of 1 × 10^6^ T2, T1, FO, or MZ B cells that were FACS purified from naïve B6 mice. Controls received PBS. Plots show skin graft survival when graft and cell transfer are performed on mice kept in (**a**) SPF facilities (n ≥ 6/group for each experiment) and (**b**) CV facilities. (n ≥ 8/group for each experiment). Mean survival in days is denoted between parentheses. Statistics were calculated by log-rank (Mantel-Cox) test + Bonferonni correction for multiple comparisons. (**C**–**G**) B cells were isolated from spleens of B6 mice maintained in SPF (**c**,**d**) or CV (E-G) facilities by magnetic sorting. B cell subsets were purified by FACS and co-cultured with negatively isolated CD4^+^ T cells and irradiated allo-DCs (25 CD4 T cells:25 B cells:1 allo-DC) for 48 hrs. PMA, Ionomycin and brefeldin A were added for the last 4 hours of culture. (**c** and **e**) Representative FACS plots of CD4^+^ T cell TNF-α expression, (**d** and **f**) summary data showing CD4^+^ T cells TNF-α expression, (**g**) summary data showing IL-10 expression by B cell subsets isolated from spleens of B6 mice housed in CV facility. Histograms display mean ± SEM, (n = 3). (**h**) Summary data showing IL-10 secretion in cultures of T cell alone and with each subset of B cells from mice treated with antibiotics (ATB) or saline solution (CV) and kept in CV facilities. Statistics were calculated by one-way ANOVA and Bonferroni post-test, ns- not significant, ^*^P < 0.05, ^**^P < 0.005, ^***^P = 0.0005.

Next, we investigated whether a particular subpopulation of B cells was responsible for prolonging skin graft survival. No differences were found in the proportions of the various B cell subsets in the spleens of mice maintained in SPF and CV facilities (Fig. S1c). To test the ability of individual B cell subsets to prolong graft survival, transitional-1 (T1), transitional-2 (T2), follicular (FO) and marginal zone (MZ) B cells were sorted by flow cytometry from the spleens of naïve C57BL/6 (B6) mice housed SPF facilities (Fig. S2) and 1 × 10^6^ cells of each subset was adoptively transferred intravenously (i.v.) to naïve B6 mice. One day following adoptive transfer B6 mice (H-2^b^) received dorsal skin transplants as described in Fig. 1. As shown in the Fig. 1a, none of the B cell subsets from mice housed in SPF conditions were able to prolong skin graft survival (Fig. 4a). We then tested whether any B cell subsets isolated from mice housed in CV facilities were able to prolong graft survival. Figure 4b shows that adoptive transfer 1 × 10^6^ T2 B cells, or T1 B cells, significantly prolonged the survival of B6-K^d^ skin grafts compared to control mice when B cell donor were from mice housed in CV conditions.

To further confirm the differences in the regulatory capacity between transitional B cells isolated from mice housed in the SPF and CV facilities, B cell subsets were tested *in vitro* for the ability to suppress allo-specific T cell activation. B cell subsets were sorted from the spleens of B6 mice, maintained in the SPF and CV facilities and co-cultured with CD4^+^ T cells stimulated by irradiated H2-K^d^ expressing allogeneic dendritic cells (allo-DCs) for 48 hours. Our results show that following stimulation of CD4^+^ T cells with allo-DC, TNF-α expression (Fig. 4c,d) and IFN-γ (Fig. S3a) by T cells and expression of CD44 and CD69 molecules (Fig. S3a) were not altered by any B cell subset from mice maintained in SPF facilities. In contrast, when B cell subsets were isolated from the spleens of B6 mice maintained in CV facilities, T2 B cells and T1 B cells suppressed TNF-α expression by CD4^+^ T cell by around 40% (although for T1 B cells the differences did not reach statistical significance), (Fig. 4e,f). The production of IFN-γ and the upregulation of CD44 and CD69 were minimal and no differences were observed (Fig. S3b). No other B cell subset suppressed allo-specific TNF-α expression by CD4^+^ T cells. In terms of the cytokines produced by the different B cell subsets derived from CV facility we observed that IL-10 expression by B cells and suppressive capacity did not correlate, as MZ B cells expressed more IL-10 than other B cell subsets even though they did not suppress T cell TNF-α expression (Fig. 4g). A similar IL-10 profile was observed in the different B cell subsets derived from mice house in the SPF facility (data not shown). Therefore, we suggest that IL-10 produced by B cells is only partially involved to supress the T cell response as we have previously demonstrated^20^.

Next, we evaluated whether treatment of CV housed mice with antibiotics altered the *in vitro* suppressive capacity of the different B cell subsets. Figure 4h shows that IL-10 levels were drastically reduced in the supernatants of co-cultures between CD4^+^ T cells, from spleen of mice housed in SPF facilities, and T2, or T1, B cells isolated from CV housed mice treated with antibiotics compared with B cells isolated from control untreated mice (Fig. 4h). These results correlated with the reduction in the IL-10^+^ B cells found *ex-vivo* and in the percentages of suppression *in vitro* (Fig. 3e,f). These results suggest that antibiotic treatment reduces the ability of T2 and T1 B cells to induce regulatory cytokine production in interactions with CD4^+^ T cells.

## Discussion

In this study we have demonstrated that B cells acquire the capacity to prolong skin graft survival when the mice are housed under non-hygienic conditions. We have shown that B cell regulatory function and ability to prolong allograft survival correlate with the presence of gut microbiota and thus, antibiotics-treatment abolished the regulatory function of B cells. In addition, we observed that mice kept in CV facilities had an increase in memory T cells in the LN(s), a higher numbers of GC B cell and an apparent bias towards an anti-inflammatory microenvironment. Furthermore, we observed that B cells derived from IL-10^−/−^ mice did not prolong skin graft survival. Finally, we provide evidence that among the different B cell subsets the regulatory function is in the transitional B cell subsets.

It has been well demonstrated by us and others that adoptive transfer of Tregs has the capacity to induce transplant tolerance^27^,^36^,^37^,^38^,^39^. We have previously shown that the adoptive transfer of 5 × 10^6^ Tregs with indirect specificity for the graft, conferred by TCR transduction, to C57BL/6 mice receiving a MHC-class I mismatched skin grafts has the capacity to prolong skin graft survival for 9 days^27^. In the experimental model presented here using the same strain combination, the adoptive transfer of 10^7^ autologous B cells, and as few as 1 × 10^6^ T2 and T1 B cells, was capable of inducing a significant prolongation of B6-K^d^ skin graft survival compared to controls (>4 days). These results suggest that the adoptive transfer of B cells is almost as powerful as the adoptive transfer of alloantigen specific Tregs for prolonging skin transplant survival.

Recently, it has been reported that the adoptive transfer of 10^7^ TIM-1^+^ B cells was capable of prolonging the survival of pancreatic islet allografts in B cell deficient (JHD mice) transplant recipients^14^. In this model TIM-1^+^ B cells were generated by either treatment of donor mice with anti-TIM-1 antibody or by the presence of an allograft, however the adoptive transfer of TIM-1^+^ B cells isolated from naïve mice did not prolong islet graft survival^14^. It is possible that if TIM-1^+^ B cells were isolated from naïve mice kept in non-sterile conditions then they may possess regulatory potential without prior *in vivo* activation. Low level activator signals associated with the presence of commensal microbiota may play a similar role to the inflammation associated with the presence of an allograft and license B cells to achieve a regulatory potential. It has been previously reported that B cells must be activated to have full regulatory potential^21^,^26^.

The importance of environmental microorganisms for B cell function *in vivo* was demonstrated by a recent study where B cells isolated from the spleens and PPs of mice kept in a CV facility secreted higher levels of antibody than mice kept in germ free facilities^34^. Similarly, we observed that housing mice under non-hygienic conditions resulted in increased number of GC B cells, changes to the cytokine production of B cells following restimulation *in vitro* (Fig. 2e). Thus, our data supports the idea that the presence of environmental microorganisms can shape the murine B cell response.

The importance of microbiota for the development of B cells with regulatory function in our study agrees with the previous reports of Shimomura *et al.* and Rosser *et al.*^25^,^28^ linked peritoneal CD5^−^ B-1 B cell mediated protection from colitis to the housing conditions of the mice, while Rosser *et al.* recently demonstrated that the presence of gut microbiota is crucial for the development of splenic regulatory T2-MZP Bregs^25^,^28^. Here we extend these results and demonstrate that the gut microbiota are also crucial for generating T2 regulatory B cell ability to suppress allo-immune responses. Interestingly, Shimomura *et al.* found no role for IL-10 production by their proposed peritoneal regulatory B cells while Rosser *et al.* found it was necessary for splenic regulatory B cell function.

Previous studies in autoimmune models have provided evidence for the role of IL-10 in the function of regulatory B cells. In our study, we found no difference in IL-10 production between B cells isolated from CV and SPF mice *in vitro* nor did we find a correlation between B cell subset IL-10 production and regulatory capacity. However, although frequency of IL10+ B cells from CV and SPF mice are the same, the percentages of TNF-α^+^ B cells are higher in cells derived from the SPF compared to CV facilities, suggesting it is the ratio of pro-inflammatory to anti-inflammatory cytokine production that may be important in deciding whether B cell transfer delays graft rejection or not (Fig. 1f). This hypothesis is supported by the recent results in human renal transplant recipients in which the authors demonstrate that the ratio of IL-10 and TNF-α expression is more important than IL-10 expression alone for measuring the function of regulatory B cells^40^.

A direct role for IL-10 in this skin transplant model was demonstrated by the inability of B cells isolated from IL-10^−/−^ mice, or mice pretreated with blocking anti-IL-10R antibody, to prolong skin graft survival on adoptive transfer (Fig. 1d,f). These results suggest that, while IL-10 expression alone is not sufficient to identify regulatory B cells, its production may potentially be important for their development in naïve mice and their subsequent ability to prolong skin graft survival. In transplant models the role played by B cell IL-10 production is still controversial^9^,^10^,^14^,^41^. While the importance of IL-10 expression to the ability of TIM-1^+^ B cells to prolong pancreatic islet allograft survival has been demonstrated^14^ other studies have been unable to identify a role for B cell IL-10 production in transplant tolerance^9^,^10^,^41^. In humans, three recent publications that reported an association of B cells with tolerance to kidney transplants in humans did not report an association with IL-10 mRNA transcription^9^,^10^,^41^. In mice, studies of the capacity of anti-CD45RB monotherapy to induce tolerance to cardiac allografts reported that, while this tolerance protocol was dependent on the presence of B cells, IL-10 production by B cells was counter-regulatory and that IL-10 neutralization improved graft outcome^42^.

Some of the signals that mediate the effect of microbiota on regulatory B cell function have been recently identified^25^. IL-1β and IL-6 produced by monocytes and dendritic cells has been suggested to be responsible for microbiota mediated regulatory B cell induction; the presence of gut microbiota no longer has the ability to generate T2-MZP Bregs *in vivo* if the B cells specifically do not express either of these receptors^25^. TLR ligation by microbial antigens for instance may also play a role; it has been reported that TLR agonists can directly stimulate B cells to produce cytokines and antibodies^43^, and TLR ligands have been suggested to be the major stimuli involved in the activation of B10 regulatory B cells^21^,^26^. However, in our study LPS-treatment of B cells derived from the SPF facility did not induce regulatory function in B cells. It is possible that other cellular target of LPS, or other factors together with LPS, can contribute to the acquisition by B cells of regulatory function.

In conclusion, the data presented here indicate that bacteria could shape B cell regulatory function. This is consistent with the ‘hygiene hypothesis’ that links ability to tolerate allergens to prior exposure to a spectrum of environmental antigens^33^. We suggest that ability to tolerate allografts may be similarly enhanced by regulatory B cells that have been conditioned by bacterial antigens or metabolites derived from the intestinal microbiota. More work is needed to understand the mechanisms behind the outcome observed in our model.

## Material & Methods

### Ethics Statement

These studies were approved and conducted in accredited facilities in accordance with The Home Office UK Animals (Scientific Procedures) Act 1986 (Home Office license number PPL 70/7302).

### Mice

Wild type (WT) C57BL/6 (H-2^b^) mice and BALB/c (H-2^d^) mice were purchased from Harlan Laboratory. B6K^d^ mice were a generous gift from Dr. R Pat Bucy (University of Alabama at Birmingham, Birmingham, AL, USA). IL-10^−/−^ mice were kindly provided by Anne O’Garra (NIMR, London). Mice were maintained in specific pathogen free (SPF) or conventional (CV) facilities. In some expreriments, mice house in CV facility were injected every 3 days for 2-3 weeks with 300 μg/mouse with anti-mouseIL-10R (clone 1B1.3A, BioXcell). In other experiments, mice kept in CV facilities were treated with antibiotics as described^44^. Alterations are as noted: ampicillin (0.5 g/l; Roche, UK), vancomycin (0.5 gm/l; Sigma, UK), neomycin sulfate (0.5 g/l; Sigma, UK), and metronidazole (0.5 g/l; Sigma) were dissolved in filtered drinking water and fluid intake was monitored. These studies and experimental protocols were approved and conducted in accredited facilities in accordance with The Home Office UK (Scientific Procedures) Act 1986 (Home Office license number PPL 70/7302) and King’s College London.

### Antibodies and Flow Cytometry

All FACS antibodies were purchased from eBioscience. For immunohistochemistry FITC-conjugated anti-B220 mAb and biotinylated anti-IgD were purchased from eBiosciences streptavidin-conjugated alexafluor594 was purchased from Molecular Probes. Surface stainings were performed as previously described^45^. For intracellular staining, cells were stained then fixed/permeabilized according to the eBioscience protocol. Permeabilized cells were incubated in Permwash™ with anti-IL10, anti-TNF, or appropriate isotype control for 30 mins at 4 °C (with blocking anti-CD16/32). Intracellular staining for FOXP3 was performed with eBioscience FOXP3 kit according to manufacturer’s instructions. For the detection of intracellular cytokines PMA (50 ng/ml), ionomycin (1 μM) and brefeldin A were added for 5 hrs before staining. Cells were acquired using LSRII or Fortessa flow cytometers (BD Biosciences). Flow Jo software was used for analysis.

### Purification of B cells, B cell subsets and CD4^+^ T cells

B cells were purified from the spleens of IL-10^−/−^ or C57BL/6 mice by negative selection using CD43 microbeads (Miltenyi Biotech, 130-049-801) according to manufacturer’s instructions. Purified splenic B cells were stained with anti-CD21, anti-CD24 anti-CD23. DAPI was added to exclude dead cells. Cells were sorted by BD FACSAria II (BD Biosciences). CD4^+^ T cells were isolated from the spleens of C57BL/6 mice using Invitrogen Dynabeads® Untouched™ Mouse CD4 Cells kits, according to manufacturer’s instructions (Invitrogen, 11416D).

### B cell stimulation with LPS

B cells from spleen were culture at a density of 10^6^/mL with or without LPS from *E. coli* serotype EH100 (TLR grade) (ALX-581-01-L002) at a final concentration of 2 μg/ml for 16 hrs at 37 °C, 5%CO_2_. Cells were then washed and resuspend at a concentration of 5 × 10^7^ ml in saline. Mice were injected i.v. with 10^7^ cells in 200 μl.

### DC generation

DCs were generated from bone marrow as previously described^45^. One day before using the DCs, 100 ng/mL LPS (E. coli, Enzo Life Sciences, UK) was added to induce maturation. At day 7 of culture, DCs were collected, washed three times, and irradiated.

### Skin transplants

Donor tail skin grafts were performed and monitored as previously described^45^. Anti-CD8 antibody (clone YTS169, 250 μg/injection/mouse) was injected i.p. at day-1 and day 1 after skin graft, and weekly thereafter.

### *In vitro* suppression assays

Isolated CD4^+^ T cells were co-cultured with irradiated allo-DCs alone at a ratio of 25 T cells:1 DC, or with B cells and allo-DCs at a ratio of 25 T cells: 25 B cells: 1 DC. Cells were cultured for 48 hrs at 37 °C in RPMI in 96 well plates (1.5–2 × 10^5^ cells/well).

### Immunohistochemistry

Tissues were embedded in OCT compound (TissueTek) and snap-frozen in liquid nitrogen. Frozen sections (7 μm) were fixed in 50% acetone for 30 seconds and 100% acetone for 5 min at 4 °C and then air-dried at room temperature. The tissue sections were blocked with normal horse serum in PBS (5%) for 15 min. The sections were stained for 30–60 mins with FITC-conjugated anti-B220 mAb and biotinylated anti-IgD, followed by streptavidin-conjugated alexafluor594. The slides were then washed in PBS, mounted with fluorescent mounting medium (Vector), visualised on a fluorescent microscope and analysed using microscope manager and Image J. All images are 10× magnification.

### Antibiotic Treatment

Mice were treated as described^44^. Alterations are as noted: ampicillin (0.5 g/l; Roche, UK), vancomycin (0.5 gm/l; Sigma, UK), neomycin sulfate (0.5 g/l; Sigma, UK), and metronidazole (0.5 g/l; Sigma) were dissolved in filtered drinking water and fluid intake was monitored.

### Fluorescent *in-situ* hybridization (FISH-Flow)

Stool samples were collected and stored at −80 °C. Fluorescent *in situ* hybridisation (FISH) combined with flow cytometry was performed as previously described with minor modifications^46^. Faeces (0.05 g) were homogenised in 0.5 ml of sterile phosphate-buffered saline (PBS, 130 mM NaCl, 3 mM NaH_2_PO_4_ 2H_2_O, 7 mM Na_2_HPO_4_ 12H_2_O, pH 7.2) with 5 glass beads in the Tissue Lyser (Qiagen, UK) for 5 min at 25 Hz. One volume of the suspension was added to 3 volumes of 4% paraformaldehyde (PFA) in PBS. After overnight fixation at 4 °C, the fixed suspension in PFA was stored at −80 °C until hybridisation. The EUB 338 probe was used as the positive control probe, the NON 338 probe as the negative control and specific probes for identification of sub-groups (Fig. S4). All probes were covalently linked at their 5′ end either to fluorescein isothiocyanate (FITC) or to the sulfoindocyanine dye indodicarbocyanine (Cy5) as indicated (Fig. S4). Samples were acquired by flow cytometry (HTS Fortessa, Becton Dickinson, USA). Subsequent analyses were conducted using FlowJo software (Tree Star, USA).

### Statistical analysis

Comparisons between groups were performed using T-test for two groups, or one-way ANOVA and a Dunnett or Bonferroni post-test for more than two groups. Log Rank (Mantel-Cox) tests were used for graft survival curves. Analyses were performed using GraphPad Prism software.

## Additional Information

**How to cite this article**: Alhabbab, R. *et al.* Diversity of gut microflora is required for the generation of B cell with regulatory properties in a skin graft model. *Sci. Rep.* **5**, 11554; doi: 10.1038/srep11554 (2015).

## Supplementary Material

Supplementary Information
