# Author Correction: Diversity of gut microflora is required for the generation of B cell with regulatory properties in a skin graft model

**DOI:** 10.1038/s41598-020-62381-5

**Published:** 2020-04-06

**Authors:** R. Alhabbab, P. Blair, R. Elgueta, E. Stolarczyk, E. Marks, P. D. Becker, K. Ratnasothy, L. Smyth, N. Safinia, E. Sharif-Paghaleh, S. O’Connell, R. J. Noelle, G. M. Lord, J. K. Howard, J. Spencer, R. I. Lechler, G. Lombardi

**Affiliations:** 1 Centre of Immunology, Division of Applied Medical Sciences, King Abdulaziz University, Jeddah, Kingdom of Saudi Arabia; 2 Medical Research Council Centre for Transplantation, King’s College London, King’s Health Partners, Guy’s Hospital, London, SE1 9RT UK; 3 Division of Diabetes and Nutritional Sciences, King’s College London, Guy’s Hospital, London, SE1 9RT UK; 4 Peter Gorer Department of Immunobiology, King’s College London, Guy’s Hospital, London, SE1 9RT UK

Correction to: *Scientific Reports* 10.1038/srep11554, published online 25 June 2015

This Article contains an error in the affiliations for R. Alhabbab, where ‘Centre of Immunology, Division of Applied Medical Sciences, King Abdulaziz University, KSA’ is incorrectly listed as a current address. The correct affiliations appear below:

Centre of Immunology, Division of Applied Medical Sciences, King Abdulaziz University, KSA.

Medical Research Council Centre for Transplantation, King’s College London, King’s Health Partners, Guy’s Hospital, London, SE1 9RT, UK.

