# Supplementary material for: Diversity of gut microflora is required for the generation of B cell with regulatory properties in a skin graft model
Source: Sci Rep. 2015 Jun 25;5:11554. doi: 10.1038/srep11554 (PMC4479822; doi:10.1038/srep11554)
Supplement: Supplementary Information [file srep11554-s1.pdf]

Diversity of gut microflora is required for the generation of B cell with regulatory properties in a skin graft model

R. Alhabbab[1,4†], P. Blair[1†], R. Elgueta[1†], E. Stolarczyk[2], E. Marks[1], P. D. Becker[1], K. Ratnasothy[1], L. Smyth[1], N. Safinia[1], E. Sharif-Paghaleh[1], S. O'Connell[1], R. J. Noelle[1], G. M. Lord[1], J. K. Howard[2], J. Spencer[3], R. I. Lechler[1,§], and G. Lombardi[1,§,*]

Alhabbab et al. Supplemental Figure 1

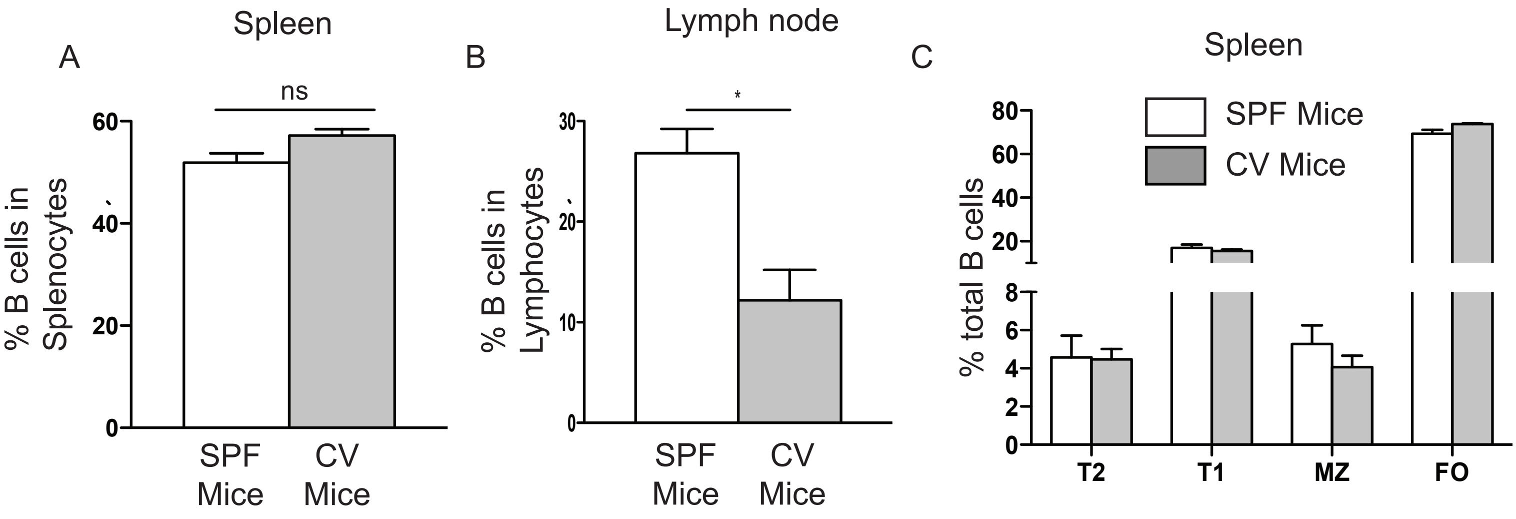

Supplemental Figure 1 Differences in total B cells and B cell subsets in the spleens and LNs of mice maintained in the CV and SPF facilities. Spleens and LNs were harvested from B6 mice (6 weeks old) that had been maintained in SPF or CV facilities. Splenocytes and lymphocytes were analysed by flow cytometry for total B cells and B cell populations by using antibodies against CD19, CD21, CD23 and CD24. Histograms displaying mean percentages + SEM of (A) total B cells as a percentage of splenocytes, (B) total B cells as a percentage of lymphocytes in lymph node. (C) splenic B cell subsets as a percentage of total B cells, n=3. Statistics were calculated by t test, * $P<0.05$.

Alhabbab et al. Supplemental Figure 2

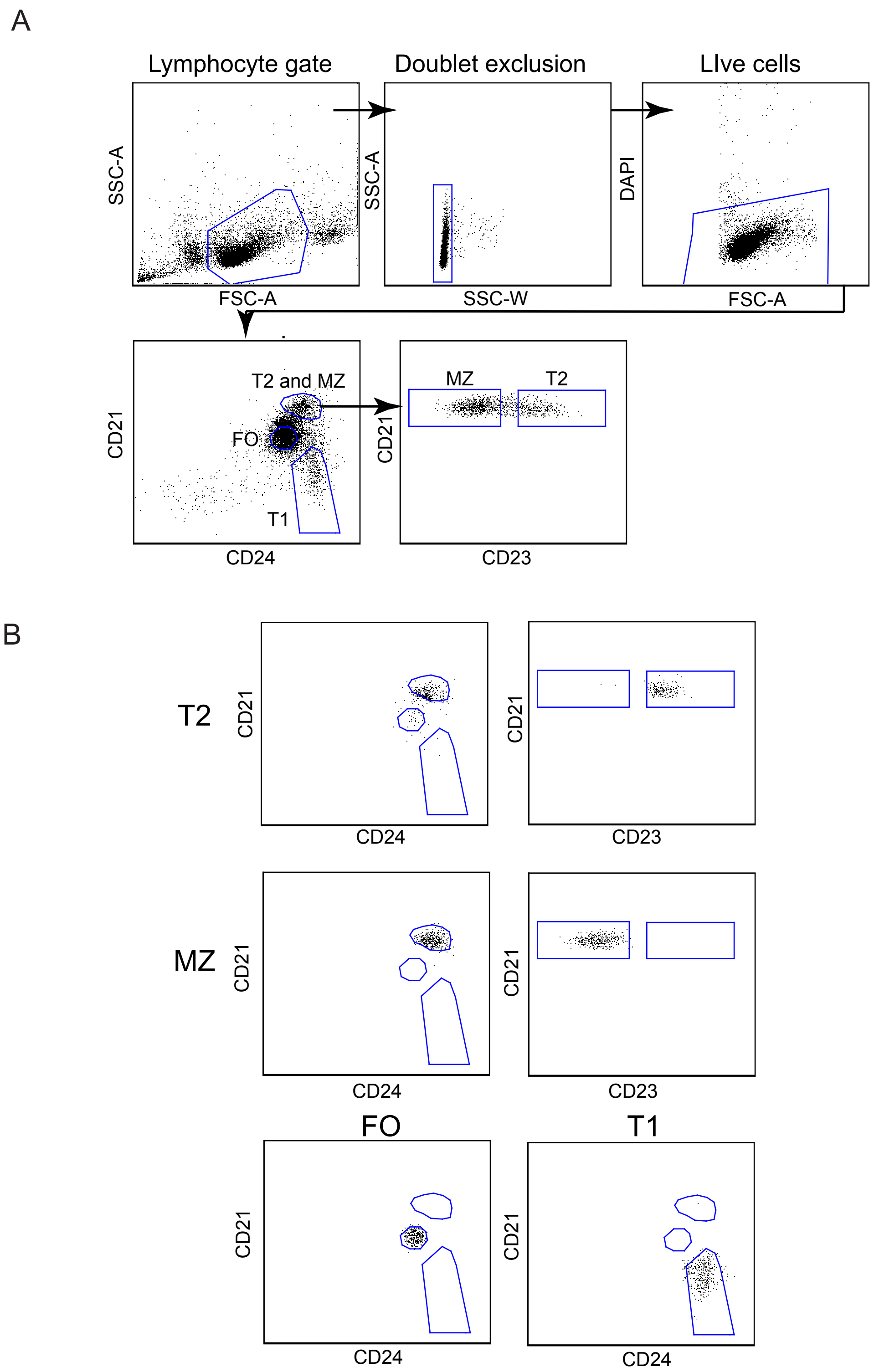

Supplemental Figure 2 B cell subset sorting strategy. B cells were isolated from spleens of naïve B6 mice by magnetic sorting and stained with antibodies against CD21, CD24, CD23 and with DAPI. Transitional-1 (T1), transitional-2 (T2), marginal zone (MZ) and follicular (FO) B cells were purified by BD FACSAria II. Representative FACS plots of, (A) Presort gating strategy, (B) B cell subsets post-sorting.

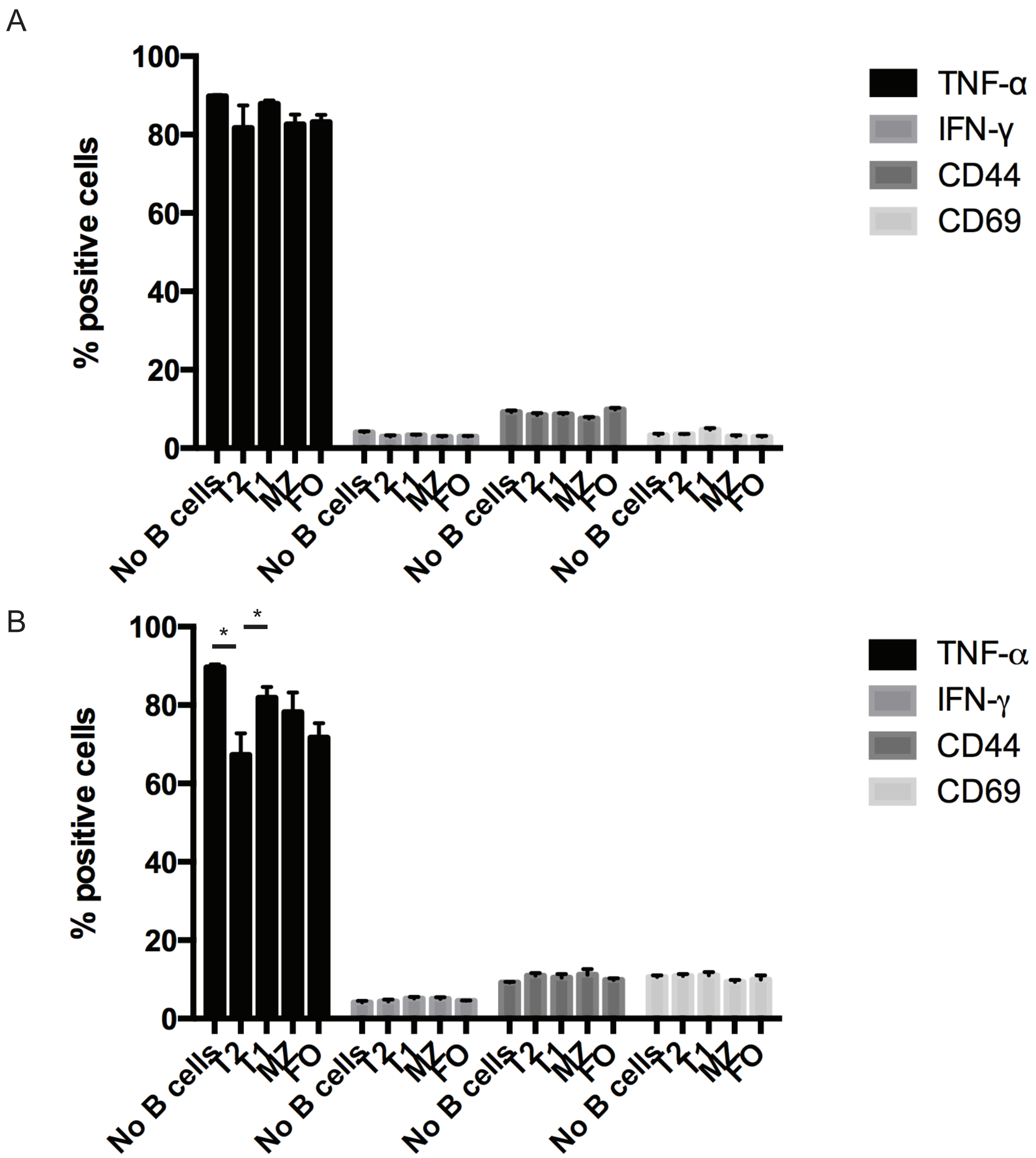

Supplemental Figure 3: B cells isolated from mice maintained in CV facilities suppress TNF-α expression by CD4+ T cells. B cells were isolated from spleens of B6 mice maintained in SPF (A) or CV (B) facilities by magnetic sorting. B cell subsets were purified by FACS and co-cultured with negatively isolated CD4+ T cells and irradiated allo-DCs from mice housed in SPF facilities (25 CD4 T cells:25 B cells:1 allo-DC) for 48hrs. PMA, Ionomycin and brefeldin A were added for the last 4 hours of culture. Summary data showing TNF-α, IFN-γ, CD44 and CD69 expression on CD4+ T cells. As a control no B cells were added to the co-cultures. Graph display mean±SEM, (n=3). Statistics were calculated by one-way ANOVA and Bonferroni post-tests. *$p<0.05$.

Table 1: Panel of group-specific probes (16S rRNA) selected to analyze the fecal microbiota composition by FISH-Flow

Probe	Sequence	Target	Label 5'	Working concentration	References
EUB 338	GCTGCCTCCCGTAGGAGT	Domain Bacteria	FITC	2ng/µl	**1**
NON 338	ACATCCTACGGGAGGC	Negative probe	Cy5	2ng/µl (control of Bac 303 and Erec 482) and 20ng/µl (control of Lab 158 and Bif 164)	**2**
Bac 303	CCAATGTGGGGGACCTT	Bacteroides	Cy5	2ng/µl	**3**
Erec 482	GCTTCTTAGTCARGTACCG	Clostridium coccoides–Eubacterium rectale	Cy5	2ng/µl	**4**
Lab 158	GGTATTAGCAYCTGTTTCCA	Lactobacillus-Streptococcus group	Cy5	20ng/µl	**5**
Bif 164	CATCCGGCATTACCACCC	Bifidobacterium	Cy5	20ng/µl	**6**

1 Amann RI, Binder BJ, Olson RJ, Chisholm SW, Devereux R, Stahl DA. Combination of 16S rRNA-targeted oligonucleotide probes with flow cytometry for analyzing mixed microbial populations. Appl Environ Microbiol. 1990 Jun;56(6):1919-25. PubMed PMID: 2200342. Epub 1990/06/01. eng.

2 Wallner G, Amann R, Beisker W. Optimizing fluorescent in situ hybridization with rRNA-targeted oligonucleotide probes for flow cytometric identification of microorganisms. Cytometry. 1993;14(2):136-43. PubMed PMID: 7679962. Epub 1993/01/01. eng.

3 Manz W, Amann R, Ludwig W, Vancanneyt M, Schleifer KH. Application of a suite of 16S rRNA-specific oligonucleotide probes designed to investigate bacteria of the phylum cytophaga-flavobacter-bacteroides in the natural environment. Microbiology. 1996 May;142 (Pt 5):1097-106. PubMed PMID: 8704951. Epub 1996/05/01. eng.

4. Franks AH, Harmsen HJ, Raangs GC, Jansen GJ, Schut F, Welling GW. Variations of bacterial populations in human feces measured by fluorescent in situ hybridization with group-specific 16S rRNA-targeted oligonucleotide probes. Appl Environ Microbiol. 1998 Sep;64(9):3336-45. PubMed PMID: 9726880. Epub 1998/09/03. eng.

5 Harmsen HJ, Kengen HM, Akkermans AD, Stams AJ, de Vos WM. Detection and localization of syntrophic propionate-oxidizing bacteria in granular sludge by in situ hybridization using 16S rRNA-based oligonucleotide probes. Appl Environ Microbiol. 1996 May;62(5):1656-63. PubMed PMID: 8633864. Epub 1996/05/01. eng.

6 Langendijk PS, Schut F, Jansen GJ, Raangs GC, Kamphuis GR, Wilkinson MH, et al. Quantitative fluorescence in situ hybridization of Bifidobacterium spp. with genus-specific 16S rRNA-targeted probes and its application in fecal samples. Appl Environ Microbiol. 1995 Aug;61(8):3069-75. PubMed PMID: 7487040. Epub 1995/08/01. eng.
